# Nationwide shifts in the double burden of overweight and underweight in Vietnamese adults in 2000 and 2005: two national nutrition surveys

**DOI:** 10.1186/1471-2458-11-62

**Published:** 2011-01-30

**Authors:** Do TP Ha, Edith JM Feskens, Paul Deurenberg, Le B Mai, Nguyen C Khan, Frans J Kok

**Affiliations:** 1 Community Nutrition Department, National Institute of Nutrition, Hanoi, Vietnam; 2 Human Nutrition Division, Wageningen University, Wageningen, the Netherlands; 3 Nutrition Consultant, Singapore; 4 Food Administration, Ministry of Health, Vietnam

## Abstract

**Background:**

In developing countries, overweight prevalence is increasing while underweight prevalence is still high. This situation is known as the double nutrition burden. Both underweight and overweight are related to increased risk of chronic non-communicable diseases, reduced well-being and quality of life. This study aims to compare the prevalence of overweight and underweight among Vietnamese adults in 2000 and 2005.

**Methods:**

The study was based on two nationally representative surveys, the National Nutrition Survey 2000 (14,452 subjects) and the National Adult Obesity Survey 2005 (17,213 subjects). Adults aged 25-64 years were sampled to be nationally representative. Multiple multinomial logistic regression analysis was used to investigate the association of underweight and overweight with socio-economic indicators.

**Results:**

The distribution of BMI across the population and population groups indicated a shift towards higher BMI levels in 2005 as compared to 2000. The nationwide prevalence of overweight (BMI ≥ 25 kg/m^2^) and obesity (BMI ≥ 30 kg/m^2^) was 6.6% and 0.4% respectively in 2005, almost twice the rates of 2000 (3.5% and 0.2%). Using the Asian BMI cut-off of 23 kg/m^2 ^the overweight prevalence was 16.3% in 2005 and 11.7% in 2000. In contrast, the underweight prevalence (BMI < 18.5 kg/m^2^) of 20.9% in 2005 was lower than the rate of 25.0% in 2000. Women were more likely to be both underweight and overweight as compared to men in both 2000 and 2005. Urban residents were more likely to be overweight and less likely to be underweight as compared to rural residents in both years. The shifts from underweight to overweight were clearer among the higher food expenditure levels.

**Conclusions:**

The double nutrition burden was clearly present in Vietnam. The distribution of BMI across the population groups generally indicated a shift towards higher BMI levels in 2005 as compared to 2000. The prevalence of overweight was increased while the declined level of undernutrition was still high in 2005. The shifts of underweight to overweight were most obvious among population groups with higher food expenditure levels.

## Background

In developing countries the prevalence of overweight is increasing, while underweight prevalence is still high. This situation is known as the double burden of malnutrition [1]. Although the underweight prevalence is decreasing it is still high, between 20% and 50%, in countries such as India, Bangladesh, China, Philippines, Thailand and Vietnam. Undernutrition is associated with increased comorbidities such as osteoporosis and diabetes [2,3]. Underweight reproductive-age women have increased risks of infertility, pregnancy complications, and giving birth to stunted and thin babies who are more likely to suffer from the diet-related diseases that were formerly thought to be associated with increasing affluence, such as diabetes, coronary heart disease and hypertension. Malnourished adults have lower work output in physical labour, earn less at work, are less productive, and are less likely to be hired as daily wage labour compared to better-nourished adults [3].

On the other hand overweight and obesity are increasing, particularly in urban areas [4,5]. Overweight and obesity are regarded as severe risk factors for a number of non-communicable diseases such as type 2 diabetes, cardiovascular disease and several forms of cancer [6]. It was estimated that only 20% of chronic disease deaths occurred in high income countries - while 80% occurred in low and middle income countries, where most of the world's population lives [7]. Controlling, or better even, preventing overweight and obesity is regarded essential in the prevention of non-communicable chronic diseases. Generally, it is clear that double burden of nutrition needs to be investigated and controlled.

Vietnam is a developing country located in South East Asia. The country covers an area of 331,000 square km, of which three fourths are highlands and mountains. With its natural characteristics, Vietnam is divided into eight ecological regions. The population increased from 77.6 million in 2000 to 83 million in 2005, with a decrease of its inhabitants living in rural areas from 76% to 73% [8]. Over the period of 1993-2004, Vietnam was considered as one of the best performers in the world in terms of economic growth [9]. Parallel with socio-economic development, the dietary pattern and lifestyle of the Vietnamese population has been changing. The composition of the diet shifted to lower amounts of starchy staples and higher amounts of proteins and lipids (meat, fish, other protein-rich or high fat foods) [10]. The higher level of industrialization and modernization brings a lifestyle with less physical activity and more sedentary habits. As a result, Vietnam is now in a period of nutrition transition and faces a double burden of nutritional problems, both underweight and overweight [11,12], similar as to other countries in the area.

In Vietnam, the problem of underweight or overweight have been studied mainly in specific groups such as children and reproductive age women and with small scale studies [13]. Surveys on overweight and obesity have reported a range of adult overweight prevalences from 8% to 18% in the period 1999-2003 [14-16]. These findings do not allow drawing nationwide conclusions on nutritional status of adult population. Two studies documented differences in the period 1992-2002, observing a reduction in underweight in adults aged 18-65 years, from 31.2% in 1992 to 24.3% in 2002, and an increase in overweight from 2% to 5.2% over this ten-year period [11,17]. The current paper aims to compare the prevalence of overweight and underweight among Vietnamese adults in 2000 and 2005, using the most updated nationally representative data of the National Nutrition Survey in 2000 (NNS 2000) and the National Adult Obesity Survey in 2005 (NAOS 2005). Given the strong economic development we expect to observe considerable changes.

## Methods

### Subjects and sampling

For this paper, data from the National Nutrition Survey in 2000 (NNS 2000) and the National Adult Obesity Survey in 2005 (NAOS 2005) were used. Both surveys used a stratified two-stage sample design.

In short, the sample was selected from the 3% household sample frame of the National Population and Housing Census (NPHC) in 1999, which was stratified by ecological regions, provinces and urban-rural area. The sample selection was done independently within each of eight ecological regions. In the first stage, 30 clusters were selected with the systematic random sampling based on the 3% sample frame. In the second sampling stage the sampling was different between NNS 2000 and NAOS 2005. In the NNS 2000, one third of the households in each cluster was selected by systematic sampling and all household members were invited for data collection. In the NAOS 2005, in each cluster 72 subjects aged 25-64 years were selected randomly and equally based on 4 age groups (25-34, 35-44, 45-54 and 55-64 years) and both genders. For both surveys, selected households (NNS 2000) or subjects (NAOS 2005) who were not able to present at the surveys were replaced by other randomly selected households or subjects (with the same gender, age group of the same cluster) based on the available sample frame of each cluster, as recommended [18]. Data were available of 14,452 (NNS 2000) and 17,213 (NAOS 2005) adults, aged 25 to 64 years.

### Nutritional status measurement and assessment

Height and weight were measured by standard methods using calibrated instruments [18]. Height was measured by using wooden stadiometer with accuracy at 1 mm. Weight was measured by using SECA electronic scale with accuracy at 100 g. Body mass index (BMI, kg/m^2^) was calculated as weight (in kg) divided by body height (in m) squared. Underweight, normal weight, overweight and obesity were classified using BMI cut-off points classified by the WHO: < 18.5 kg/m^2 ^is underweight, 18.5-24.9 kg/m^2 ^is normal weight, ≥25 kg/m^2 ^is overweight and ≥ 30 kg/m^2 ^is obesity [18]. Additional cut-off points suggested to use for Asian populations were also used, i.e.: ≥23 kg/m^2 ^for overweight and ≥27.5 kg/m^2 ^for obesity [19].

### Statistical analysis

Data analysis was done using SPSS version 15.0 (SPSS Inc. Chicago, Ill). In order to ensure a fully national representative reporting of the result, both datasets were analyzed by the complex sample procedures using weighing factors based on the population structure stratified by ecological region, gender, age group and urban-rural area in 1999 (NPHC, 1999) and in 2004 (Census, 2004) for the datasets of NNS 2000 and NAOS 2005, respectively.

Pregnant women and under-12 month lactating mothers as well as subjects with extreme and/or implausible height, weight or BMI were excluded. In the dataset of NNS 2000, data of 324 pregnant and lactating women and 15 subjects with extreme height or weight were excluded from the analysis. In NAOS 2005 pregnant and under-12 month lactating women were excluded in the sampling procedure. In the data analysis phase 32 subjects with extreme height or weight were excluded. Data were therefore available on 14,452 (NNS 2000) and 17,213 (NAOS 2005) adults, aged 25 to 64 years.

The data are presented as percentages with 95% confidence interval (95% CI), stratified by ecological region, area of residence, age group, gender, education level and food expenditure level. Multiple multinomial logistic regression, logistic regression for polytomous instead of dichotomous outcomes, was used to investigate the relationship of socio-economic factors with both underweight and overweight Results were presented as odds ratios (OR) with 95% CI comparing overweight or underweight to normal weight. Variables which were available in both datasets were included in the regression models, i.e. age group, gender, area of residence, education level and food expenditure (as income proxy). The food expenditure variable was the average monthly food expenditure per capita (in Vietnam currency). Food expenditure was categorized into 5 levels based on percentiles with level 1 is the lowest and level 5 is the highest. The education levels ranged from illiterate, literate, primary school, secondary school, high school and higher education. The estimation of number of overweight or underweight population was the products of the prevalence of overweight or underweight × number of population aged 25-64 years old.

### Ethical consideration

The study was approved by the Ethical committee of the National Institute of Nutrition - Vietnam Ministry of Health. Participants were asked for agreement to participate in the surveys prior to the data collection. Full access to the datasets was approved by the Vietnam National Institute of Nutrition.

## Results

### Trend of BMI distribution

The distribution of BMI across the whole population and population groups generally indicated a shift towards higher BMI levels in 2005 as compared to 2000 (Table 1). Nationwide, the overall prevalence of overweight (BMI ≥ 25 kg/m^2^) and obesity (BMI ≥ 30 kg/m^2^) were 6.6% (95% CI: 5.9-7.4%) and 0.4% (95% CI: 0.3-0.6%) in 2005, which were almost twice the rates in 2000, 3.5% (95% CI: 3.0-4.0%) and 0.2% (95% CI: 0.1-0.2%). When applying the suggested cut-off points for Asia, the prevalence of overweight (BMI ≥ 23 kg/m^2^) and obesity (BMI ≥ 27.5 kg/m^2^) were 16.3% (95% CI: 15.1-17.5%) and 1.7% (1.4-2.0%) in 2005, in comparison with 11.7% (95% CI: 10.6-12.9%) and 0.9% (95% CI: 0.7-1.1%) in 2000. Based on these data, the average annual increase in overweight prevalence was 0.62%/year. In contrast to the shift to higher overweight and obesity prevalence, the underweight prevalence (BMI < 18.5 kg/m^2^) showed a lower level in as 2005 compared to 2000, i.e. 20.9% (95% CI: 19.6-22.1%) compared to 25.0% (95% CI: 23.5-26.5%), respectively. The average annual decrease in underweight prevalence was 0.82%/year. Table 1 also shows that both the prevalence of overweight and underweight were higher in women as compared to men in both years.

**Table 1:** BMI distribution (%, 95% CI) in adults aged 25-64 years in 2000 and 2005 by gender

	N	BMI < 18.5 kg/m^2^	**BMI **≥ **23 kg/m^2^**	**BMI **≥ **25 kg/m^2^**	**BMI **≥ **27.5 kg/m^2^**	**BMI **≥ **30 kg/m^2^**
	**National Nutrition Survey 2000**					
Nationwide	14452	25.0 (23.5-26.5)	11.7 (10.6-12.9)	3.5 (3.0-4.0)	0.9 (0.7-1.1)	0.12 (0.1-0.2)
Male	7044	22.0 (20.4-23.7)	9.6 (8.4-10.9)	2.8 (2.2-3.4)	0.7 (0.4-1.0)	0.1 (0.0-0.2)
Female	7408	27.9 (26.0-29.8)	13.7 (12.3-15.3)	5.5 (4.8-6.5)	1.6 (1.2-2.0)	0.2 (0.1-0.4)
	**National Adult Obesity Survey 2005**					
Nationwide	17213	20.9 (19.6-22.1)	16.3 (15.1-17.5)	6.6 (5.9-7.4)	1.7 (1.4-2.0)	0.4 (0.3-0.6)
Male	8483	19.9 (18.4-21-4)	14.5 (13.2-16.0)	5.3 (4.6-6.1)	1.2 (0.9-1.6)	0.4 (0.2-0.6)
Female	8730	21.9 (20.4-23.5)	18.1 (16.7-19.7)	8.0 (7.1-9.0)	2.2 (1.8-2.7)	0.5 (0.3-0.7)

**Figure 1:**
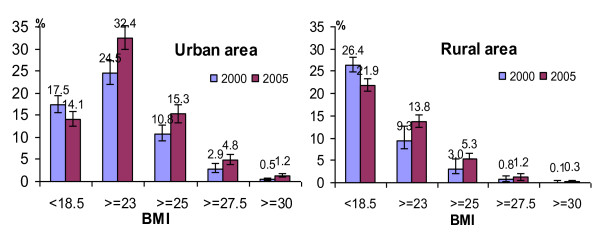
**BMI distribution (%; 95% CI) in adults aged 25-64 years in 2000 and 2005 by area of residence**.

Concerning area of residence, the shift from underweight to overweight was observed in both urban and rural areas (Figure 1). The overweight and obesity prevalence was higher in 2005 as compared to 2000 in both rural and urban areas with, as expected, the highest prevalence in the urban area. The underweight prevalence was lower in 2005 as compared to 2000 in both areas of residence, but always higher in rural area compared to urban area in both years.

### Double burden of overweight and underweight by various sub-groups

**Figure 2:**
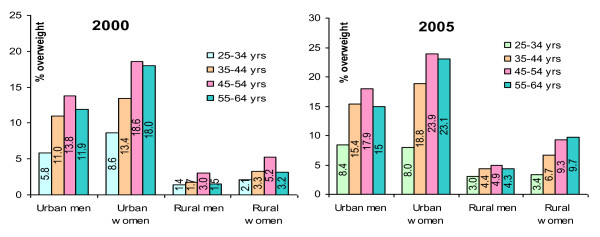
**Prevalence of overweight (BMI ≥ 25 kg/m**^**2**^**) in 2000 and 2005 by age group, sex and area of residence**.

Figure 2 shows the prevalence of overweight across different subgroups of gender, area of residence and age in 2000 and 2005. The general trend of higher rates in 2005 was observed in all subgroups (men and women, urban and rural areas and different age groups). In both areas and genders the prevalence of overweight was generally higher with higher age, with the highest prevalence for age group 45-54 years in both 2000 and 2005.

**Figure 3:**
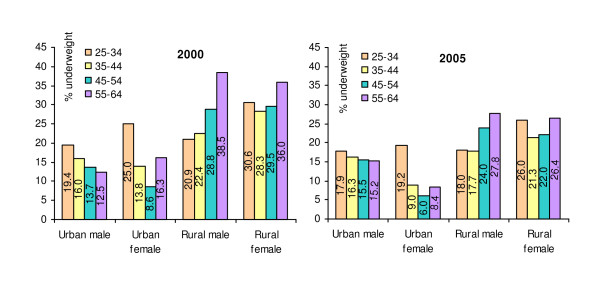
**Prevalence of underweight (BMI < 18.5 kg/m**^**2**^**) in 2000 and 2005 by age group, sex and area of residence**.

The underweight prevalence by different subgroups is presented in Figure 3. The highest underweight prevalence was observed in the youngest men and women (25-34 years old) in the urban area in contrast to the oldest men and women (55-64 years old) in the rural area, although in 2005 the differences were less pronounced as compared to 2000. Among urban men, the underweight prevalence by age was about similar in both years, while among rural men underweight prevalence was lower in 2005 as compared to 2000, similar to the prevalence among urban and rural women.

Generally, underweight was less frequent among the older compared to the younger age groups among urban men, while it was more frequent among higher age groups among rural men. Among urban women, underweight was most prevalent among the youngest group aged 25-34 years. Among rural women, the youngest and the oldest groups were more frequently underweight as compared to the other age-groups in both years.

**Figure 4:**
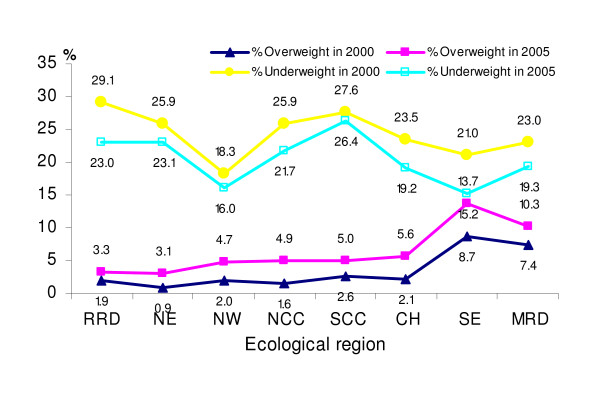
**Prevalence of underweight (BMI < 18.5 kg/m**^**2**^**) and overweight (BMI ≥ 25 kg/m**^**2**^**) in 2000 and 2005 by ecological region**. RRD: Red river delta, NE: Northeast, NW: Northwest, NCC: North Central Coast, SCC: South Central Coast, WH: West Highland, SE: Southeast, MRD: Mekong river delta.

The double burden of overweight and underweight across eight ecological regions in 2000 and 2005 is presented in Figure 4. In all ecological regions, the prevalence of overweight was higher in 2005 as compared to 2000. The pattern of overweight across the regions was similar in both 2000 and 2005. The highest prevalence was found in the South-East and Mekong river delta regions and the lowest prevalence was seen in the North-East region, while the remaining regions did not differ much from each other. The pattern regarding underweight was also similar in both 2000 and 2005. The highest prevalence of underweight was observed in the Red river delta and South Central Coast, while the lowest was found in North-West and South-East regions in both years.

**Figure 5:**
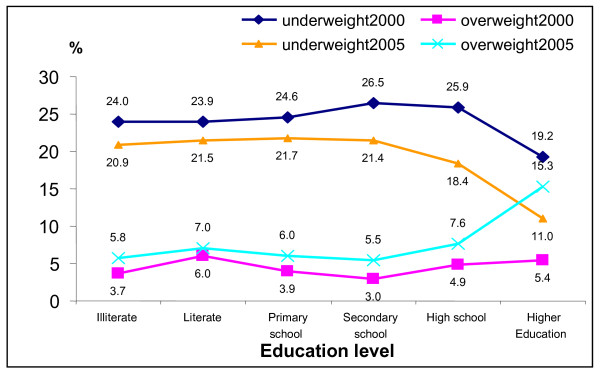
**Prevalence of underweight and overweight by education level in 2000 and 2005**.

Figure 5 shows that there were quite similar patterns regarding the prevalence of overweight and underweight according to education levels in 2000 and 2005. The underweight prevalence was about similar for the four lower education levels and gradually reduced from the of Secondary school level to the Higher education level. This contrast in underweight between lower and higher education levels was larger in 2005 as compared to 2000. The prevalence of overweight gradually increased from the levels of lower education to Secondary school and quickly raised the highest rate at the Higher education level. Also this contrast was larger in 2005 as compared to 2000.

**Figure 6:**
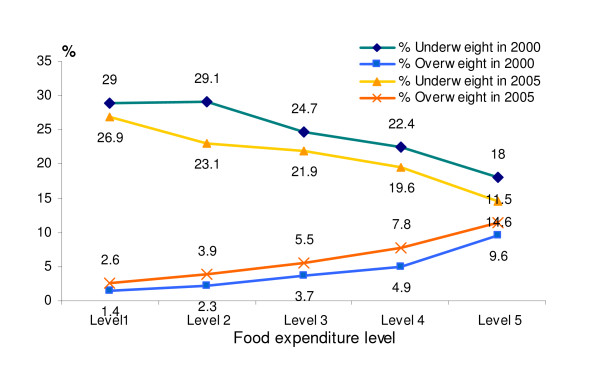
**Prevalence of underweight and overweight by food expenditure level in 2000 and 2005**.

The prevalence of overweight and underweight by food expenditure levels are presented in Figure 6. At higher categories of the food expenditure, the prevalence of overweight was higher and the prevalence of underweight was lower, in both years.

### Multiple logistic regression analysis

Table 2 presents the independent association of several socio-economic factors with underweight and overweight in both 2000 and 2005, using multiple multinomial logistic regression analysis.

**Table 2:** Relationship of selected socio-economic factors with overweight and underweight in 2000 and 2005

	Adjusted OR (95% CI)					
	
	2000			2005		
	N	Underweight	Overweight	N	Underweight	Overweight
**Age group**						
25-34	5,024	1.00	1.00	4,335	1.00	1.00
35-44	5,147	0.95 (0.85-1.07)	1.56 (1.11-2.18)*	4,354	0.85 (0.74-0.97)*	1.95 (1.48-2.57)*
45-54	2,780	1.15 (1.00-1.31)*	2.26 (1.62-3.15)*	4,325	1.10 (0.95-1.27)	2.79 (2.16-3.61)*
55-64	1,501	1.65 (1.38-1.97)*	1.5 (0.92-2.34)	4,199	1.34 (1.15-1.56)*	2.66 (1.99-3.56)*
**Gender**						
Male	7,044	1.00	1.00	8483	1.00	1.00
Female	7,408	1.46 (1.31-1.63)*	2.03 (1.64-2.52)*	8730	1.16 (1.03-1.31)*	1.53(1.29-1.83)*
**Area of residence**						
Rural	10,616	1.00	1.00	13,145	1.00	1.00
Urban	3,836	0.76 (0.63-0.92)*	2.39 (1.79-3.19)*	4,068	0.80 (0.64-0.99)*	2.08 (1.60-2.72)*
**Education level**						
Illiterate	1,239	1.00	1.00	1,675	1.00	1.00
Literate	2,675	1.13 (0.88-1.44)	1.29 (0.82-2.05)	3,139	1.24 (0.93-1.64)	0.73 (0.53-1.01)
Primary school	4,175	1.27 (0.99-1.64)	0.81 (0.5-1.31)	3,151	1.38 (1.03-1.87)*	0.68 (0.45-1.00)
Secondary school	3,920	1.43 (1.07-1.91)*	0.6 (0.37-0.97)*	5,783	1.36 (1.01-1.84)*	0.59 (0.41-0.85)*
High school	1,582	1.62 (1.17-2.26)*	0.69 (0.42-1.13)	2,017	1.30 (0.93-1.81)	0.70 (0.47-1.04)
Higher education	809	1.28 (0.88-1.88)	0.44 (0.25-0.79)*	447	1.07 (0.63-1.08)	0.90 (0.55-1.49)
**Food expenditure**						
Level 1	2,895	1.00	1.00	2,987	1.00	1.00
Level 2	2,889	1.00 (0.86-1.16)	1.72 (1.06-2.80)*	2,936	0.82 (0.69-0.98)*	1.49 (1.01-2.19)*
Level 3	2,892	0.82 (0.69-0.97)*	2.45 (1.58-3.79)*	2,946	0.75 (0.62-0.89)*	2.09 (1.41-3.11)*
Level 4	2,876	0.75 (0.63-0.89)*	2.94 (1.87-4.62)*	2,943	0.71 (0.59-0.85)*	3.00 (1.98-4.53)*
Level 5	2,890	0.64 (0.54-0.76)*	4.74 (2.95-7.61)*	2,947	0.55 (0.44-0.69)*	3.49 (2.32-5.24)*

OR: odds ratio; CI: Confidence interval

* Significant at p < 0.001.

Women were always more likely to be both underweight (OR = 1.4 and OR = 1.16 in 2000 and 2005, respectively) or overweight (OR = 2.03 and OR = 1.53 resp.). After adjustment for education level and food expenditure level, as proxy for income, urban residents were always less likely to be underweight (OR = 0.76 in 2000 and 0.80 in 2005) and more likely to be overweight (OR = 2.39 and OR = 2.08). Food expenditure level, as proxy for income, was independently associated with underweight and overweight in both years. The higher the food expenditure, and thus the higher the income, the more overweight (OR = 1.72 to 4.74 and OR = 1.49 to 3.49) and the less underweight (OR = 1.00 to 0.64 and OR = 0.82 to 0.55) was observed. Regarding educational level the results were different from the unadjusted analyses as shown in Figure 5. After adjustment for food expenditure level, area, age and gender, the higher education groups were generally more likely to be underweight and less likely to be overweight as compared to the lowest education level. This was mainly due to the association with food expenditure level, which was more strongly related to underweight and overweight than education.

## Discussion

This study shows that the prevalence of overweight (BMI ≥ 25 kg/m^2^) among Vietnamese adults aged 25-64 years in 2005 was almost twice as compared to 2000. The estimated average increase in prevalence of overweight amounted to 0.6%/year, which was almost twice as that of 0.3%/year over the period of 1992-2002 [11]. Based on our data, the estimated number of subjects aged 25-64 years with overweight in 2005 amounted to about 2.6 million (BMI≥ 25 kg/m^2^) or 6.5 million (BMI≥ 23 kg/m^2^).

The pattern of overweight and obesity prevalence across population groups defined by age, gender and/or areas of residence were similar between the two periods (1992-2002 and 2000-2005), with a higher prevalence among women, urban residents, and older age-groups. The higher estimated increase in the nationwide prevalence of overweight and obesity and the bigger differences between urban and rural areas observed in 1992-2002 and 2000-2005 highlights the increasing problem of overweight in Vietnam, particularly in the urban areas.

The increasing trend of overweight and obesity is not only observed in Vietnam but also in other countries in the Asian region as well as worldwide. Fortunately, the magnitude of the problem in Vietnam is still much less than in many of these countries, such as 29% (1996-1997) in Hongkong [20] and 26.7% (1998) in Korea [21], which might be due to lower level of economic development in Vietnam. In Thailand, the National Health Examination Survey II showed a prevalence of overweight (BMI ≥ 25 kg/m^2^) in adults aged 20-59 years of 28%, with highest values in women (33.9%) and in the urban population (34.8%) [22]. The problem of overweight and obesity is also rapidly increasing in China in all gender and age groups and in geographical areas, particularly in the urban area, with overall reported prevalence rates of 15% in 1992 and 22% in 2002 [23]. The higher prevalence of overweight and obesity among women and urban residents in Thailand and China were similar to the situation found in the present study.

The prevalence of urban overweight (BMI ≥ 25 kg/m^2^) in our nation-wide samples is in concordance with previous smaller studies done in Hanoi and Ho Chi Minh City which reported overweight in 17.2% to 18.5% of adults aged 20-60 years [14-16]. In the past, the percentage of overweight in the adults, was also higher in urban areas than in rural areas (4.8% vs. 1.2% in 1998 and 9.6% vs. 3.5% in 2002) [17]. This higher prevalence in the urban area may be explained by the faster economic growth. Over the period 1993-2004 Vietnam was considered to be one of the best performers in the world in terms of economic growth [9]. As a result, poverty rates were halved in the same period. The general poverty rates decreased from 37% to 20%, while the food poverty rate went down from 13% to 7%. In parallel with the economic growth, the urbanization went up and the rate of urbanization is expected to remain above 3% per annum until 2020. It was estimated that the urban area accounted for 70% of the growth while containing only 25% of the population [24]. We used food expenditure as a proxy indicator of income, and this was indeed also associated with higher overweight rates. But independent of food expenditure and other demographic factor overweight was still twice as high in urban areas compared to rural areas. In addition to higher income, urbanization has brought changes in lifestyle and food consumption habit which may also contribute to the higher prevalence of overweight. In urban areas of developing countries, food scarcity may no longer be the driving force behind energy intake. Instead, the availability of cheap, energy-dense foods (including those from street vendors and fast food restaurants) may facilitate the consumption of more calories. Widespread access to television would favour an indoor, sedentary lifestyle, further reducing the average daily energy expenditure [25]. Those changes lead to an obesogenic environment.

The prevalences of overweight and obesity differed across the eight ecological regions but were all higher in 2005 as compared to 2000. There were several reasons for these differences and changes, but they were likely to be closely related to socio-economic status. Household poverty status significantly influences food consumption and food patterns [10]. There were considerable disparities in regional poverty and poverty reduction [9]. The South East region had the lowest poverty rate, which reduced from 12% in 1998 to 5% in 2004, and the same region was shown to have the highest and fastest increase in the prevalence of overweight and obesity. The Northern mountains (North East and North West), the North Central Coast and the Central Highland all have high poverty rates (50% and above in 1998 and still over 30% in 2004) and accordingly have lower prevalence rates of overweight and obesity.

In contrast with the increasing problem of overnutrition, undernutrition showed a decreasing trend. The estimated average annual reduction rate was 0.8%/year in the period 2000-2005, after an earlier reduction of underweight in adults from 31.2% in 1992 to 24.3% in 2002 [11]. This reduction is probably thanks to the economic development and the considerable achievement in nutrition policy and intervention in Vietnam [26]. In our study, food expenditure level was inversely associated with underweight. However, despite substantial improvements in rural living standards, poverty levels were still remarkably high in the rural area [9] in addition to the high prevalence of underweight. In the past decades, the available data showed that the prevalence of underweight and stunting among children aged under 5 years were very high, e.g. 51.5% and 59.7% in 1985, 44.9% and 46.9% in 1994, 31.9% and 34.8% in 2001 [26]. In the earlier decades, a similar or even worse situation probably existed. This early childhood malnutrition situation maycontribute to the adult overweight nowadays [27]. Maternal and child malnutrition control should be strengthened to reduce child undernutrition in order to prevent adulthood underweight and overweight, as well as the related chronic diseases.

In terms of age, the highest prevalence of overweight was observed in the age category of 45-54 years. Only for rural women, the age pattern was somewhat different, with the highest prevalence observed in the oldest category (55-64 yrs) in 2005. The general pattern of overweight and obesity by age agrees with survey findings from other Asian countries [28,29]. The prevalence of underweight by age differed between urban and rural areas. In the urban area underweight was more prevalent in the youngest group of 25-34 years, while in the rural areas it was more prevalent in the oldest age groups. This may be explained by the immigration of young labour force from rural to urban areas due to rapid urbanization. Those young workers are mainly unskilled, having heavy manual works with low income. People who move from rural to urban areas usually lose the ability to grow their own food and thus become dependent for their calories on a cash market [25].

Interestingly, women were more likely to be both underweight and overweight as compared to men. This pattern was also reported among Indian women [30] and among Bangladesh rural and urban poor women [31]. This may reflect various disadvantages which women face, such as poor nutrition care, heavy work load, physiological characteristics, and a high prevalence of early childhood undernutrition [3]. Because of women's cyclical loss of iron and childbearing, their nutritional status is particularly vulnerable to deficiencies in diet, care, and health or sanitation services. Gender inequality exacerbates infectious diseases among the less affluent through the pathway of childhood undernutrition. At the same time, it exacerbates the new regime of chronic diseases among the relatively more affluent, possibly through a pathway that has come to be known as "the Barker hypothesis". Gender inequality thus leads to a double jeopardy, aggravating the double nutrition burden [32].

A steady shift is shown between underweight and overweight prevalence according to food expenditure, independent of age, gender and education. These findings confirm the association of economic growth with food consumption and nutritional status, particularly in developing countries where more than 50% of income is spent on food [25]. Interestingly, the observed higher prevalence of overweight and lower prevalence of underweight in the highly educated group was accounted for by the other demographic factors and food expenditure in the logistic regression analysis.

Our results indicate that it is timely and necessary to take immediate action for effective control of underweight and early prevention of the spread of overweight and obesity problem in Vietnam. However, programs and interventions should take the double nutrition burden into consideration to avoid sharpening the severity of underweight when spending efforts in reducing overweight. Appropriate interventions are needed for specific population subgroups. Some important interventions for reducing the rate of undernutrition may also be beneficial in terms of reducing the burden of obesity are promoting breast-feeding, improving nutritional status of women of reproductive age, and reducing the rates of fetal growth retardation [33] and low birth weight [25]. Improving the obesogenic environment in urban area by nutritional education, information and communication for promoting healthy eating and physical activity and monitoring food market should be intensively implemented in order to reducing underweight and preventing overweight [25]. Reducing gender inequalities should be paid attention in improving double burden of nutrition among women in particular and in the whole population in general [33]. Promoting household food production with the existing successful VAC model (i.e. the Vegetation, Aquaculture and Cattle-breeding model), particularly encouraging small-scale farmers and, especially women, to grow and utilize a wide variety of food crops toward improving household food security and dietary diversity can be an effective way for combating double nutrition burden in rural area [34].

The present study has some limitations. Data on diet, physical activity and smoking were not available for both datasets and thus could not be adjusted for. However, with data from the two largest recent nationally representative nutrition surveys, conducted by well-trained personnel according to a standardized protocol, the shift in the double burden of malnutrition was clearly demonstrated.

## Competing interests

The authors declare that they have no competing interests.

## Authors' contributions

DTPH as a principle investigator of this research have full access to all of the study data and takes responsibility for the integrity of the data and the accuracy of the data analysis; she formulated the research question, participated in data collection, conducted data analysis and wrote the paper. EJMF provided guidance in the formulation of the manuscript, critical revision of the manuscript for intellectual content and take full responsibility for the paper. PD provided methodological expertise, guidance in data analysis and critical revision of the manuscript for intellectual content. LBM was responsible for the formulation of the surveys and data collection. NCK provided guidance in formulation of the surveys, data collection and critical revision of the manuscript for intellectual content. FJK contributed guidance in the formulation of the manuscript and critical revision of the manuscript for intellectual content. All authors read and approved the final manuscript.

## Pre-publication history

The pre-publication history for this paper can be accessed here:

http://www.biomedcentral.com/1471-2458/11/62/prepub
